# Acetic Acid Enables Precise Tailoring of the Mechanical Behavior of Protein-Based Hydrogels

**DOI:** 10.1021/acs.nanolett.2c01558

**Published:** 2022-08-26

**Authors:** Marina Slawinski, Maria Kaeek, Yair Rajmiel, Luai R. Khoury

**Affiliations:** † Department of Materials Science and Engineering, Technion Israel Institute of Technology, Haifa 32000, Israel; ‡ Department of Physics, University of Wisconsin—Milwaukee, 3135 N. Maryland Ave, Milwaukee, Wisconsin 53211, United States

**Keywords:** Protein-based hydrogels, Biomaterials, Protein folding transitions, Responsive biomaterials, Dynamic hydrogels

## Abstract

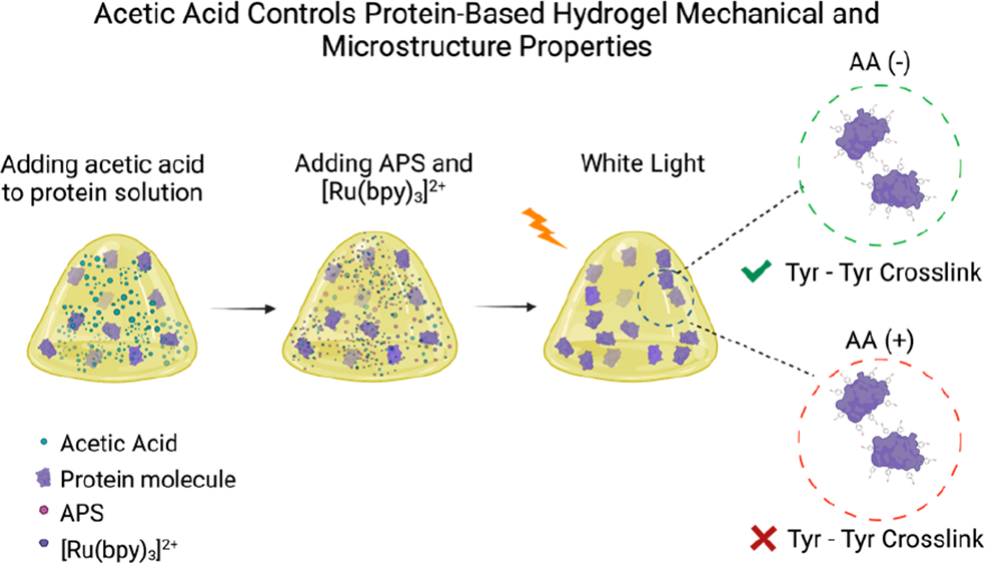

Engineering viscoelastic and biocompatible materials with tailored mechanical and microstructure properties capable of mimicking the biological stiffness (<17 kPa) or serving as bioimplants will bring protein-based hydrogels to the forefront in the biomaterials field. Here, we introduce a method that uses different concentrations of acetic acid (AA) to control the covalent tyrosine–tyrosine cross-linking interactions at the nanoscale level during protein-based hydrogel synthesis and manipulates their mechanical and microstructure properties without affecting protein concentration and (un)folding nanomechanics. We demonstrated this approach by adding AA as a precursor to the preparation buffer of a photoactivated protein-based hydrogel mixture. This strategy allowed us to synthesize hydrogels made from bovine serum albumin (BSA) and eight repeats protein L structure, with a fine-tailored wide range of stiffness (2–35 kPa). Together with protein engineering technologies, this method will open new routes in developing and investigating tunable protein-based hydrogels and extend their application toward new horizons.

Dynamic hydrogels with reversible network cross-links are promising materials in biomedical applications such as cell proliferation,^1−4^ tissue engineering,^5−7^ and shape-memory applications.^8,9^ Due to their viscoelastic behavior, these hydrogels are adaptable to external mechanical triggers such as creeping, stress-relaxation,^1,10^ self-healing,^11−14^ and energy dissipation,^15−18^ which are critical factors in determining the fate of cells in tissue regeneration processes. Several reversible cross-linking interactions have emerged in polymeric hydrogels to mimic the functional dynamics of biological tissues and enhance their mechanical properties. Hydrogen bonding,^8,19,20^ electrostatic interactions,^3,4,17,21^ and host–guest approaches^22,23^ were used to engineer hydrogels with multiple relaxation modes. In addition, metal coordination^24^ and reversible covalent cross-links^3,4,11^ were implemented to engineer and tune the viscoelastic hydrogel networks with predefined stress-relaxation time scales.

Peptide- and nonglobular-based hydrogels have attracted broad interest in biomedical applications and research. Several studies tried to utilize the intramolecular interactions of peptide chains and the precise engineering of α-helix and β-sheet structures to control and improve the mechanical and microstructure properties of peptide-based hydrogels.^25−29^ In addition, synthetic proteinaceous hydrogels constructed from nonglobular proteins such as elastin have been extensively investigated to fine-tune their mechanical and structural behavior.^30−32^ On the contrary, globular protein-based networks exhibit an inherently viscoelastic behavior with precise relaxation times, stretchability, and functionality resulting from the folding transitions of protein domains at the nanoscale level within the hydrogel matrix.^10,18,33,34^ The protein (un)folding nanomechanics were used to engineer hydrogels with shape-memory^9,35^ and morphing^36^ capabilities. Additionally, protein engineering technologies were implemented to design tandem polyprotein structures to form muscle-like biomaterials^18,33^ and self-healing protein hydrogels.^14,37^ Moreover, protein biochemical diversity and its interactions with polymers and cations have been explored to precisely tailor the mechanical behavior of hydrogel samples.^9,36^

Despite the incredible versatility in synthesis methods and applications of polymer or protein hydrogels, the formation of dynamic hydrogels with tailored stiffnesses that mimic the mechanical behavior of biological tissues (<1 to 17 kPa) remains a challenge.^38−41^ Forming hydrogels with low stiffness is possible only using low protein concentration; however, this approach produces incomplete and fast degradation of cross-linked networks. Since Fancy and Kodadek introduced the light photoactivated reaction to cross-link protein molecules through covalent tyrosine-tyrosine interactions by irradiating white light on a mixture that contains protein, ammonium persulfate (APS), and Tris(bipyridine) ruthenium(II) chloride ([(Ru(bpy)_3_]^2+^),^42^ this method has been widely used in protein-based hydrogel formation.^9,10,18,34−36,43−45^ However, the latter approach is extremely limited since a solid biomaterial can only be formed from a highly concentrated protein solution (>150 mg/mL), limiting the hydrogel’s mechanical properties and narrowing the stiffness range.^43^ On the other end, lowering the concentration will decrease the density of proteins in the cross-section area, and the force per molecule will increase, thus leading to permanent damage to the hydrogel sample under high strain or stress.^10^ For example, a bovine serum albumin (BSA) hydrogel formed with low BSA concentration (e.g., 0.7 or 1 mM) using a photoactivated reaction yielded a brittle hydrogel. Still, decreasing the stiffness of protein-based hydrogels without reducing the number of protein domains while simultaneously preserving the native folded structure and forming a reliable hydrogel is not currently possible.

Here, we introduce a significant upgrade to the conventional photoactivated reaction by adding acetic acid (AA) as a precursor in the protein hydrogel mixture to control the photochemical covalent cross-linking between exposed and adjacent tyrosine residues. We characterized the effect of AA on the protein native structure, the cross-linking density, and the mechanical and microstructure properties of the bovine serum albumin (BSA) hydrogel matrix. We found that this reaction is unique in that by increasing the AA concentration, we can controllably and precisely decrease the stiffness down to ∼1 kPa and control the microstructure and pore size of protein-based hydrogels while preserving protein native structure and concentration.

During our previous attempts to generate composite chitosan/BSA-based hydrogels from chitosan/BSA aggregates, we found that the inclusion of 1% (v/v) (175 mM) AA (pH ∼ 7.4) in the initial preparation buffer resulted in hydrogel samples that were very soft and stretchable compared to native BSA-based hydrogels. To further investigate the effects of AA as a precursor on the final properties of the hydrogel, we continued the study without chitosan, examining the differences between 2 mM BSA-based hydrogels synthesized in the presence and absence of 1% (v/v) AA.

After synthesizing 2 mM BSA hydrogels with and without AA (Figure 1a), the mechanical response of both hydrogels was measured by subjecting the samples to a force-ramp protocol with a linearly increased and decreased stress rate of 40 Pa/s (0.01 mN/s) using a custom-made force-clamp rheometer.^10,46^ The hydrogel formed from 2 mM BSA dissolved in phosphate buffer containing 1% (v/v) AA showed a significant decrease in stiffness (∼5 kPa) compared to a BSA hydrogel formed in phosphate buffer without AA (∼12 kPa), which showed similar mechanical behavior to BSA hydrogels prepared in TRIS buffer (Figure 1b).^9,10,36^ This observation intrigued us to investigate how adding AA to the phosphate buffer affects BSA structure and hydrogel physical properties.

**Figure 1:**
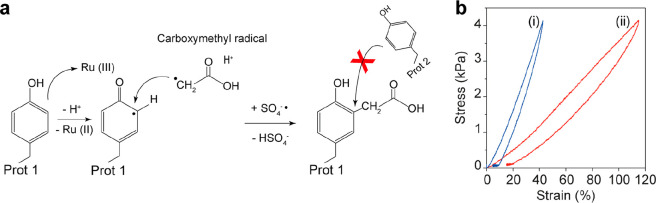
Reaction mechanism of suppressing tyrosine-tyrosine covalent cross-links during protein-based hydrogel synthesis using a photoactivated reaction in the presence of acetic acid in the preparation buffer. (a) A photoactive reaction mixture containing protein, APS, Ru (II), and acetic acid is exposed to white light at room temperature. Ru (II) is photolyzed due to exposure to visible light in the presence of APS, which leads to Ru (III) and sulfate radical generation. Then, Ru (III) oxidizes tyrosine amino acids on surrounding proteins.^42^ A nearby carboxymethyl radical (·CH_2_COOH and H atoms) produced during the photoinitiated protein cross-linking reaction may attack the radical and attach to the tyrosine amino acid, suppressing the tyrosine-tyrosine formation and decreasing the cross-linking density within the hydrogel network. In the absence of carboxymethyl radical, a covalent cross-link forms between two adjacent exposed tyrosine amino acids as previously reported, promoting the synthesis of the protein-based hydrogel.^10,42^ This scheme represents a hypothetical mechanism for the effect of AA on the tyrosine–tyrosine cross-linking mechanism. Further experiments are still needed to determine the exact mechanism. (b) Stress–strain curves of BSA-based hydrogel samples were prepared (i) in the absence of acetic acid and (ii) in the presence of acetic acid 1% (v/v) (175 mM). The hydrogel prepared in a buffer containing AA showed lower stiffness and higher extension than the hydrogel sample prepared in a buffer without AA.

The stretchability observed in the hydrogel samples prepared in the presence of AA posed two different possibilities that could cause a decrease in the hydrogel stiffness. First, adding AA into the phosphate buffer could disrupt the structural stability of the protein domains inside the matrix, causing a decrease in the stiffness of the hydrogel material. Alternatively, adding AA could decrease the cross-linking density within the hydrogel matrix, increasing stretchability and significantly decreasing the Young’s modulus. We first examined the impact of AA on the BSA native structure to reveal any modifications on the whole protein conformation. To examine whether the AA affected the folded native structure of BSA, we used a naked-eye examination technique by adding 8-anilino-1-naphthalenesulfonate (ANS) to 2 mM BSA solutions containing various AA concentrations, 0% (v/v) (0 mM), 0.5% (v/v) (87 mM), 0.75% (v/v) (131 mM), 1% (v/v) (175 mM), 1.5% (v/v) (262 mM) (Supporting Figure 1a).^47^ The insignificant change in emission measurement of ANS molecules indicates that the protein solutions preserved their 3D folded structure (Supporting Figure 1b). To further examine the secondary structure of BSA molecules pre- and postgelation with different AA concentrations, the samples were characterized by attenuated total reflectance-Fourier transform infrared (ATR-FTIR) spectroscopy. No obvious change was observed in the amide I peak with and without AA, particularly relevant to structural change in the secondary structure of BSA compared with the FTIR spectra of free BSA protein and BSA-based hydrogel samples denatured by 6 M guanidinium chloride (GuHCl) where the BSA lost the main α-helix structure (Figure 2a). These results are consistent with the circular dichroism spectroscopy measurements of BSA-based hydrogel pre- and postgelation in normal and denaturant conditions.^34^ Furthermore, the BSA secondary structures were evaluated by deconvoluting and resolving the amide I peak. The main peaks representing the protein secondary conformations are the intramolecular β-sheet (1610–1630 cm^–1^), α-helix (1648–1660 cm^–1^), and β-turn (1660–1689 cm^–1^).^48−51^ These structures were observed in BSA solution and BSA-based hydrogel samples in the presence of various AA concentrations. The extent of each secondary structure was also calculated through curve fitting of the amide I band. The results show that BSA comprises ∼20% intramolecular β-sheet, ∼70% α-helix, and 10% β-turn, which agrees with previously published results (Figure 2c).^52^

**Figure 2:**
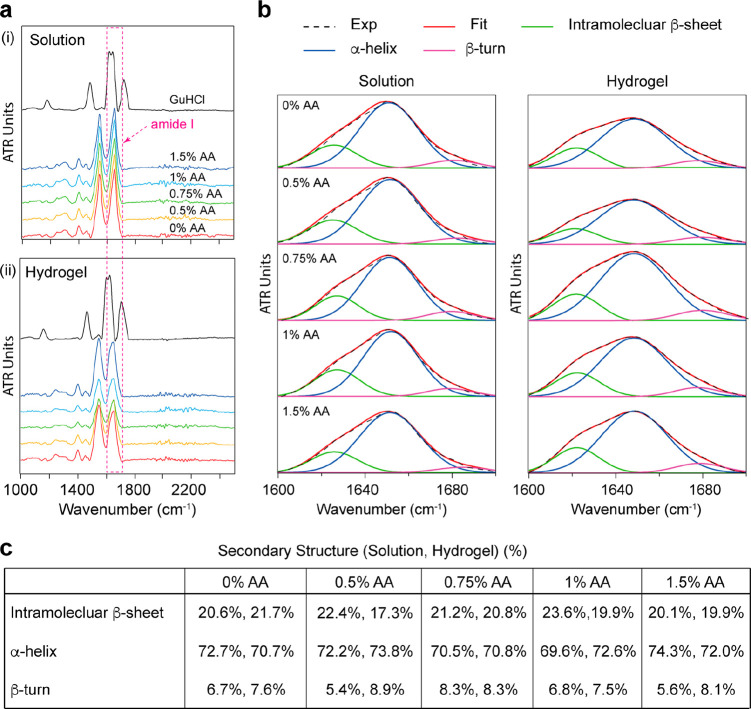
Investigating the effect of AA on the BSA native structure before and after gelation. (a) FTIR spectra of (i) 2 mM BSA solution dissolved in phosphate buffer containing various AA concentrations and in 6 M GuHCl denaturing solution; (ii) 2 mM BSA-based hydrogel samples with different AA concentrations in TRIS and 6 M GuHCl solutions. The region of the amide I band is labeled. (b) The secondary structure of BSA protein pre- and post- gelation determined by the Fourier deconvolution of each amide I band for each sample. The deconvolution of the amide I band of BSA solution and hydrogels showed that BSA protein has three secondary structures, including intramolecular β-sheet, α-helix, and β-turn. The BSA preserved its secondary structure in all solutions, which indicates that AA does not affect its native structure before and after gelation. (c) Summary of the major conformation of the secondary structure of BSA protein pre- and postgelation. The conformation content of the secondary structure was estimated by calculating the area under the curve of each peak through the amide I deconvolution.

These observations led us directly to the case that AA inhibits the formation of dityrosine cross-linking between protein domains during the photoactivation reaction. We used a hydrolysis acid protocol to investigate the effect of AA on the cross-linking density inside the hydrogel matrix.^44,53^ We found that increasing the AA concentration decreased the dityrosine bonds in each hydrogel matrix (Figure 3a), which directed us to investigate the effect of AA concentration on the mechanical behavior of BSA-based hydrogel samples. The mechanical response was investigated by subjecting the 2 mM BSA-based hydrogel samples formed with various concentrations of AA to a linearly increased/decreased stress at a rate of 40 Pa/s using our custom-made force-clamp rheometer.^10,46^ The resulting stress–strain curves assist in determining the Young’s modulus of the hydrogel samples, and the hysteresis results from folding transitions of protein domains reported the energy dissipated by each sample.^10,46^ The stiffness of the 2 mM BSA-based hydrogels decreased proportionally with increasing AA concentrations. The sample containing 1% (v/v) (262 mM) AA showed a significant decrease in stiffness down to ∼2 kPa, ∼220% stretchability, and excellent recovery (Figure 3b and c). In addition, the hydrogel samples were characterized using a cryo scanning electron microscope (cryoSEM) to investigate the effect of AA on their microstructure and morphology. The images revealed that all the samples have fibrillar mesh morphology. However, with increasing AA, we found that the average area of the pores inside the mesh increased from 0.87 ± 0.17 to 1.7 ± 2.1 μm^2^ (Figure 3d). The increase in pore density and area is consistent with swelling behavior and the decrease in cross-linking density within the hydrogel samples (Figure 3e).

**Figure 3:**
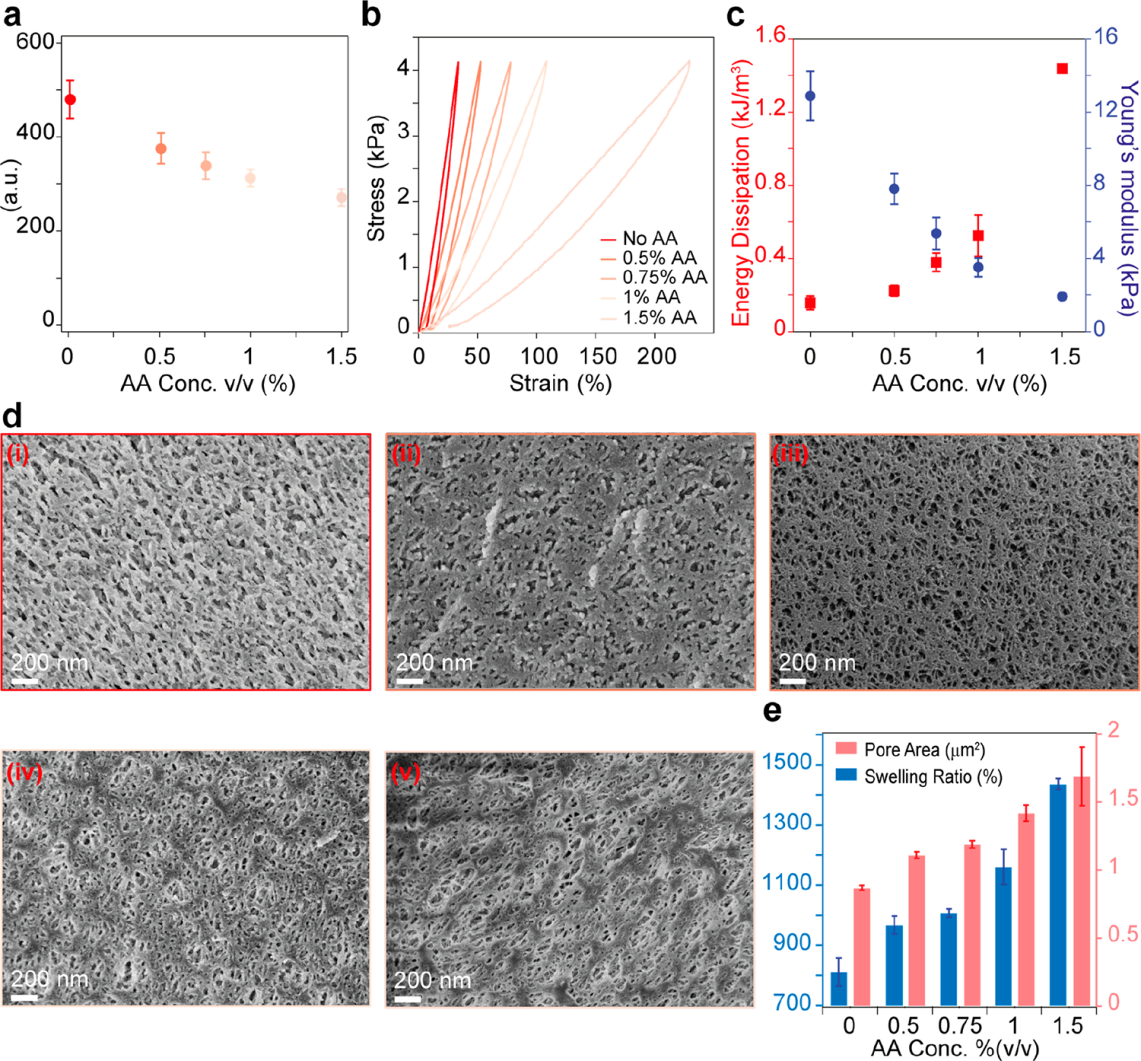
Characterizing the effect of AA on the mechanical behavior of BSA-based hydrogels. (a) Quantification of di-tyrosine bonds in BSA-based hydrogels formed with different AA concentrations. (b) Stress–strain curves of 2 mM BSA-based hydrogels with various AA concentrations. (c) Average Young’s modulus and energy dissipation vs AA concentrations as calculated from stress–strain curves. (d) CryoSEM images of BSA-based hydrogel with different AA concentrations: (i) 0% (v/v) (0 mM), (ii) 0.5% (v/v) (87 mM), (iii) 0.75% (v/v) (131 mM), (iv) 1% (v/v) (175 mM), and (v) 1.5% (v/v) (262 mM). (e) Swelling ratio and average pore-size area of the BSA-based hydrogel with different AA concentrations.

Furthermore, this platform provides us with the ability to examine the effect of BSA folding transitions and the cross-linking density on the mechanical stiffness of the hydrogel samples. Thus, we applied various loading rates on 2 mM BSA-based hydrogel samples synthesized using different (0% (v/v) (0 mM), 0.5% (v/v) (87 mM), and 0.75% (v/v) (131 mM)) AA concentrations (Supporting Figure 3). As seen in Figure 4, the Young’s modulus is a sigmoidal function of the applied loading rate. BSA-based hydrogels formed with 0.5% and 0.75% (v/v) AA showed similar behavior to samples without AA under various loading rates, while the stiffness values decreased as the AA concentrations increased (Figure 4a–c). This tendentiousness in the hydrogel behavior can be attributed to the decrease in the intramolecular and intermolecular cross-linking density inside the hydrogel samples, making the BSA domains less fettered under stress cycles thus translating into a decrease in Young’s modulus (Figure 4g–i). To support our findings, we decided to exclude the effect of protein folding transitions from hydrogel samples and examine the impact of loading rate on the mechanical behavior of the denatured BSA-based hydrogels. Thus, we applied similar loading rates to the same samples while immersed in a 6 M GuHCl solution. The GuHCl breaks the hydrogen bonds in the BSA domains, and then the denatured protein loses its 3D structure and mechanical stability. As seen in Figure 4d–f, we did not observe any change in Young’s modulus at the various loading rates for each AA concentration, and the hysteresis disappeared in all hydrogel samples during the stress/release cycles. However, the Young’s modulus for each hydrogel sample decreases with the AA concentration (Figure 4g–i). These outcomes reflect only the hydrogel samples’ elastic response, which results from the cross-linking density and corresponds to mesh size between cross-linking points^54^

**Figure 4:**
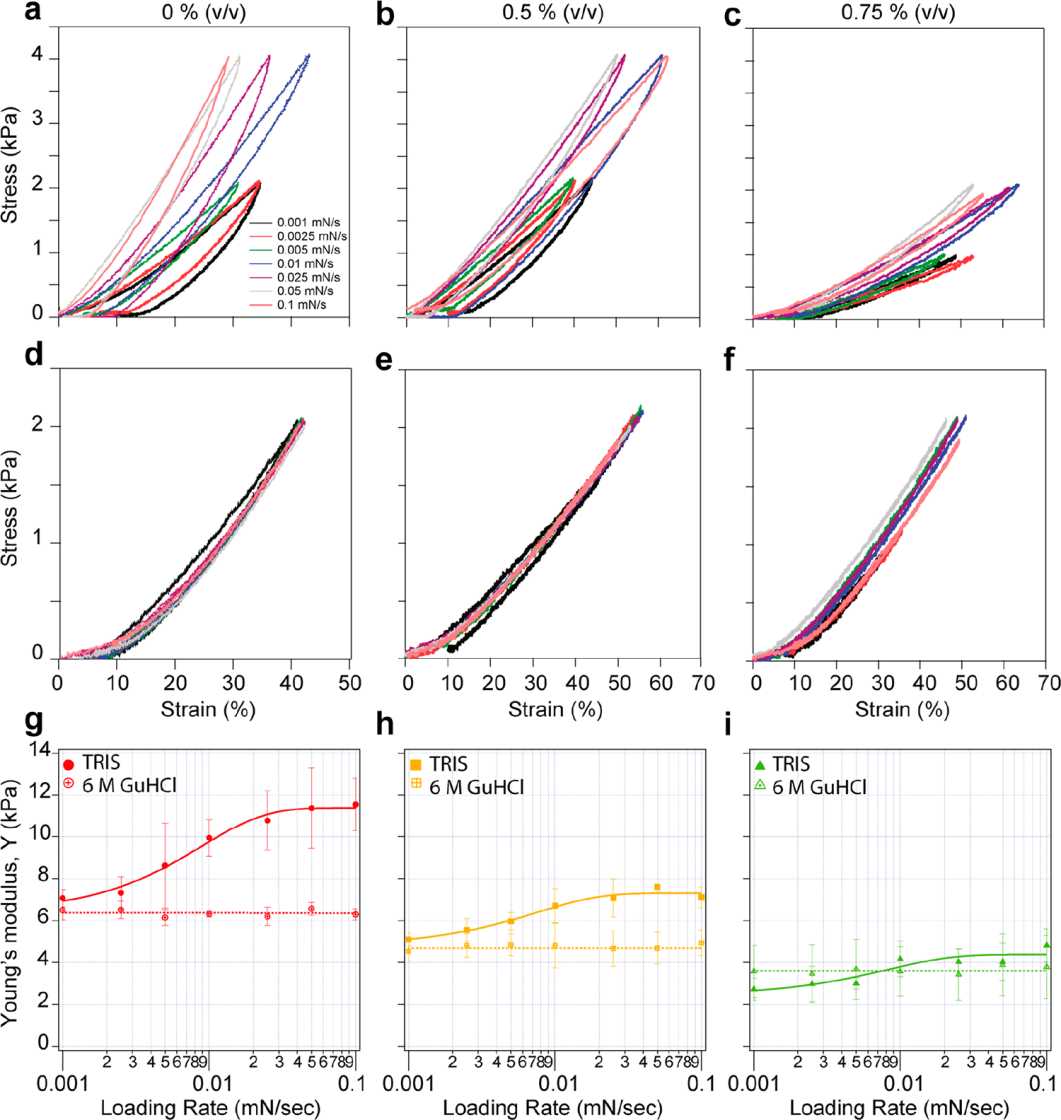
Studying the effect of cross-linking density and protein folding transitions on BSA-based hydrogel mechanical behavior. (a–c) Stress–strain curves on BSA-based hydrogels formed in the presence of various AA concentrations immersed in TRIS solution. (d–f) Stress–strain curves of BSA-based hydrogels at different AA concentrations submerged into 6 M GuHCl solution. (g–i) Average Young’s modulus of BSA-based hydrogels immersed into TRIS or 6 M GuHCl solutions as a function of loading rates. Due to the viscoelasticity resulting from the protein domains’ (un)folding mechanics, the hydrogel samples exhibit varying stiffness in response to different loading rates when characterized in TRIS solution. However, when the mechanical behavior of hydrogel samples was investigated in 6 M GuHCl, the Young’s modulus did not change at different loading rates.

To prove the feasibility of our approach toward other proteins, we engineered and synthesized an eight tandem-like protein L structure (pL-8) using a bacterial expression system. Each protein L has three tyrosine amino acids on its surface and 24 in the whole eight-domain engineered structure. Due to the high initial tyrosine density, the pL-8 showed a higher stiffness than in BSA hydrogel samples (Figure 5a). Applying the same force-clamp protocol on 1 mM pL-8 samples prepared with various concentrations of AA (0.3% (v/v) (52 mM) - 1.4% (v/v) (254 mM)) shows that increasing the AA concentration decreases the stiffness of the hydrogel samples from ∼30 kPa for native pL-8 hydrogel to ∼15 kPa with 1.4% (v/v) AA.

**Figure 5:**
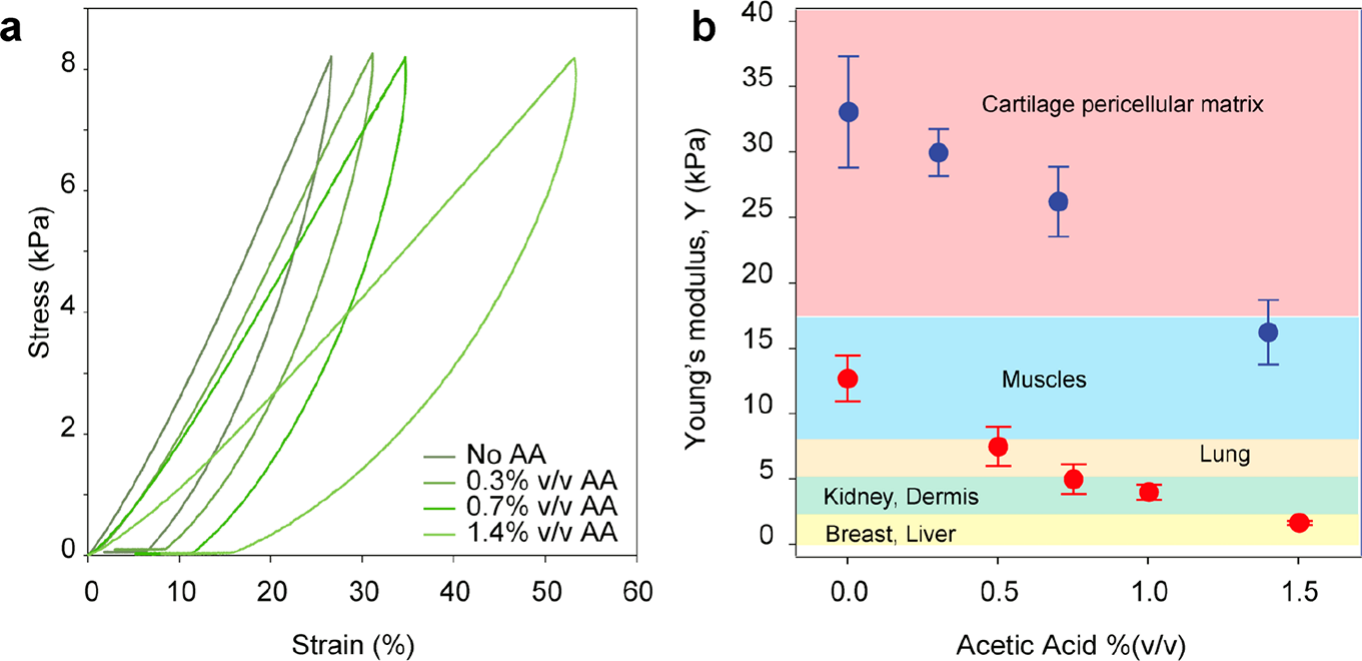
Using AA as a di-tyrosine cross-linking inhibitor to manipulate protein-based hydrogel mechanical behavior to mimic biological tissue functional dynamics. (a) Stress–strain curves of pL-8-based hydrogels with various AA concentrations. (b) Stiffness of biological tissues compared to the Young’s modulus of BSA and pL-8-based hydrogels formed in various concentrations of AA (indicated with red and blue markers, respectively).

Protein-based hydrogels are unique biomaterials as they are inherently viscoelastic and biocompatible, and engineering their protein building blocks inside the hydrogel matrix enables regulation of the material’s mechanical and structural properties at the macro and the nanoscale level. However, protein-based hydrogels still have significant drawbacks in precisely fine-tuning and mimicking the mechanical properties of biological tissues (<17 kPa).^41^ Generally, high concentrations of proteins are required (∼150 mg/mL) to generate a reliable protein-based hydrogel, which limits their mechanical tunability.^14,18,33^ Several approaches were proposed to control the mechanical properties of protein hydrogels. Nevertheless, all these strategies were focused on how to stiffen the gels. Here, adding AA as a precursor to the hydrogel buffer allows the design of protein-based hydrogels with tailored stiffness and impressive stretchability (∼200%).

By addressing the effect of AA on protein native structure using spectroscopy techniques, we found that adding AA to the buffer solution did not affect the native conformation of the protein (Supporting Figure 1). The ATR-FTIR recordings have shown that the amide I peak in all BSA solutions and BSA-based hydrogel samples did not shift compared with a denatured BSA protein (Figure 2a). The Fourier deconvolution of the amide I peak indicated that the BSA in solutions and gels has three major secondary conformations and preserves the α-helix structure (∼70%) of the BSA protein before and after gelation (Figure 2b).

Following these studies, we found that the dityrosine cross-links decreased with increasing AA concentration (Figure 3a). These findings were supported by the mechanical characterization test applied to each hydrogel sample. The decrease in Young’s modulus implies that the BSA is less constrained inside the hydrogel matrix, leading to an increase in the folding transitions in protein structure, which is translated into an increase in the dissipated energy (Figure 3c) and extreme stretchability (∼200%), particularly at 1.5% (v/v) (262 mM) AA. The increase in the average pore area can be attributed only to the decrease in cross-linking density as BSA concentration remains constant.^54^ Furthermore, we noticed that a fibrous structure characterizes the microstructure of the samples with the addition of AA. Those observations are consistent with the swelling measurement results, which show that the decrease in the cross-links is translated into less dense hydrogel allowing more water to diffuse into the hydrogel matrix (Figure 3d). This controllability over the mechanical and microstructure properties is a critical contribution to tissue engineering and drug delivery applications, which can also be modified upon need.

Tailoring the cross-linking density inside protein-based hydrogels without affecting protein structure and concentration is significant in using the hydrogel samples to study protein nanomechanics in bulk mode, as the only parameter changing is the length. The BSA-based hydrogel samples showed a mechanical response like the behavior of polyprotein chains in single-molecule force spectroscopy experiments (SMFS) under different loading rates.^55^ The latter determines a single protein’s unfolding force.^55−57^ Here, in protein-based hydrogels where millions of molecules are randomly cross-linked in the material matrix, part of the BSA domains are either fully or partially oriented with the direction of the applied force.^58^ Since the unfolding force of BSA domains, like other proteins, is correlated with mechanical and thermal forces, different loading rates provide different time scales for these forces to respond. At a high loading rate, the thermal forces have a limited time to react, leading to a higher unfolding force needed to unfold the protein domains inside the hydrogel. Thus, the protein domains are kept folded, reflecting an increase in hydrogel stiffness. On the lower end, a slow loading rate provides the protein domains enough time to allow the thermal forces to respond and simultaneously decrease the unfolding force, allowing for protein domains to unfold inside the hydrogel matrix and making the protein-based hydrogel material softer (Figure 4a−c). Moreover, characterizing the 2 mM BSA-based hydrogel samples while submerged inside the denaturing 6 M GuHCl solution (Figure 4d−f) enabled us to decouple the viscoelastic effect resulting from protein folding transitions and examine the elastic response of the hydrogel mesh, which originates only from the covalent cross-links and elastic behavior of the unfolded BSA molecules.^10^ Our results demonstrated that the elastic response of the hydrogel is affected by the cross-linking density of the dityrosine bonds. In addition, the disappearance of the hysteresis and the unchanged Young’s modulus at different loading rates for each sample provides evidence that our novel approach reduces the cross-linking density by increasing the AA concentrations. These results emphasize the significant role of protein (un)folding nanomechanics in determining protein-based hydrogels’ mechanical behavior and stiffness. The experiments conducted here prove that AA suppresses the formation of the dityrosine bond. We speculate that AA produces carboxymethyl radical (·CH_2_COOH and H atoms) during the photoactivated reaction, reducing the tyrosine radical intermediate needed to form tyrosine-tyrosine interactions.

Besides the inherent biocompatibility resulting from the protein domains, the cytotoxicity of the cross-linking reagents APS and Ru(II) was extensively assessed in various in vitro studies which found that reagents are consumed during the hydrogel formation under visible light (<10 s) and diffuse out from the network after several washes, and the concentrations drop to a level that is not toxic to various types of cells.^59−61^ In our case, exposing the hydrogel to white light for 30 min and washing it with TRIS buffer is also sufficient to remove the reagents from the hydrogel matrix (Supporting Figure 4).

Furthermore, the use of different proteins and cross-linking densities allow us to recapitulate the stiffness of various biological tissues at the macroscale level through various loading rates (Figure 5b). Our protein-based materials also have a certain degree of dynamicity, such as stress relaxation and stiffening resulting from protein folding transitions at the nanoscale level, which may be tuned to mimic the appropriate cellular behavior cells receive in the extracellular matrix environment (Figure 4).^62−67^ Moreover, the ability to preserve the protein concentration and control the stiffness of the matrix will allow us to control the material’s degradation rate to improve the postimplantation outcomes.^68^

Finally, our data suggest that this reaction allows us to control the cross-linking density and the mechanical and microstructure properties of protein-based hydrogels to fit the stiffness of various biological tissues at the macro and nanoscale level without affecting the protein structure or concentration. With an easy synthesis method, the latter insights pose these materials as an ideal substrate to investigate and be used as a fertile platform to examine protein folding transitions in a bulk approach. It is noteworthy that further studies and analyses are needed to understand the inhibition process of dityrosine formation during hydrogel synthesis. This strategy can be extended toward using different inhibitors to control the cross-linking density inside protein-based hydrogels. In addition, the significant progress in protein engineering technologies and the controlled viscoelastic properties at the macro- and nanoscale levels can place protein-based materials in the forefront line of biomaterials. Engineering protein-based hydrogels coupled with unchanged integrin-binding ligands, such as RGD, tunable mechanical behavior, inherent viscoelasticity, and biocompatibility, will be an exciting platform for 3D cell culture or as cell-laden biomaterial implants to promote tissue regeneration. Looking to the future, the developed approach will open new avenues in designing and engineering adaptive, innovative biomaterials for tissue replacement and soft-robotics applications.

## Supporting Information Available

Experimental methods; supplementary figures; additional mechanical analysis data; fluorescence images and measurements; and ATR-FTIR spectra (PDF)
