# Supplementary material for: Acetic Acid Enables Precise Tailoring of the Mechanical Behavior of Protein-Based Hydrogels
Source: Nano Lett. 2022 Aug 26;22(17):6942–50. doi: 10.1021/acs.nanolett.2c01558 (PMC9479135; doi:10.1021/acs.nanolett.2c01558)
Supplement: Supplementary file 1 — nl2c01558_si_001.pdf [file nl2c01558_si_001.pdf]

SUPPORTING INFORMATION

Acetic Acid Enables Precise Tailoring the Mechanical Behavior of Protein-Based Hydrogels

*Marina Slawinski[2], Maria Kaeek[1], Yair Rajmiel[1], Luai R. Khoury[1]**

[1] Technion Israel Institute of Technology, Department of Materials Science and Engineering, Haifa, 32000, Israel.

[2] University of Wisconsin-Milwaukee, Department of Physics, 3135 N. Maryland Ave, Milwaukee, WI 53211, US.

* Corresponding author: Luai R. Khoury, luaikh@technion.ac.il

Experimental Methods

Preparation of eight domain protein L structure (pL-8). The B1 pseudo-wild-type domain of protein L obtained from Finegoldia Magna (formerly Peptostreptococcus Magnus) was repeated eight times before being inserted in a modified pFN18a vector (Promega). Following transformation with the pFN18a vector, BLR(DE3) competent cells were grown to OD600 = 0.6 at 37°C in LB buffer in the presence of carbenicillin 50 μg/mL, and expression was induced with 1 mM Isopropyl β-D-1-thiogalactopyranosid (IPTG) overnight at 25°C. Cells were then pelleted

and re-suspended in elution/ washing (E/W) buffer (NaH_2PO_4 50 mM, NaCl 300 mM, DTT 1mM, glycerol 5% v/v, pH 7.0) and lysed with lysozyme, DNase, and RNase in the presence of protease inhibitors, followed by sonication. After cell lysis, the soluble protein fraction was passed through a chemical affinity purification NiNTA column, then eluted with E/W buffer containing 250 mM Imidazole. We used size exclusion chromatography to extract the exact pL-8 structure (Akta GE, elution in HEPES 50mM, NaCl 150 mM, pH 7.2 buffer). Then, we concentrated the pL-8 to the final concentration of 2 mM using protein concentrator columns.

Protein-based hydrogel synthesis. Phosphate buffer solutions containing NaH_2PO_4 ~ 10mM, NaCl ~ 150 mM, with different AA concentrations (0.25 – 1.5 % (v/v)) were first prepared and then balanced to pH ~ 7.4 using KOH (1 M) solution. Then, the bovine serum albumin (BSA) or p-L8 protein were dissolved in these solutions at 2- and 1-mM concentrations, respectively. The protein solution, APS, and $[Ru(bpy)_3]^{2+}$ were mixed at a volume ratio of 15:1:1. Subsequently, the mixture was centrifuged to remove air bubbles and then loaded into a transparent Teflon tube, as reported previously.[10,35] The Teflon tube containing the hydrogel mixture was placed under white light for 30 minutes at room temperature (RT). Protein-based hydrogel samples without AA were synthesized as reported in our previous study.[10,35] Afterward, the hydrogel sample was removed from the tube and immersed in TRIS solution (20 mM Tris, 150 mM NaCl, pH~ 7.4) to equilibrate.

Fluorescence measurements of AA effect on BSA stability. Triplicates containing 200 μL of 2 mM BSA at various AA concentrations (0 - 1.5% (v/v)) were mixed with 1 μL of 20 mM 8-aniline-1-naphthalenesulfonate (ANS) solution (6 μM). Following 10 min of incubation at RT, the fluorescence emission intensity was recorded at 480 nm using the infinite M200 spectrophotometer (TECAN) plate-reader.

ATR-FTIR Measurements. FTIR spectra of BSA solution and hydrogel samples were recorded using Nicolet iS50 FT-IR in attenuated total reflection (ATR) mode equipped with a round Diamond, Type IIa crystal. 16 scans were recorded for each sample measurement with a nominal resolution of 8 cm^{-1}. The content of three main secondary structures of BSA, including intramolecular β-sheets (1610-1630 cm^{-1}), α-helix (1648-1660 cm^{-1}), and β-turns (1660-1689 cm^{-1}), was analyzed by the spectral deconvolution of the Amide I band (1600 to 1700 cm^{-1}), according to previous studies.[42,44,54,55]

Quantifying the effect of AA on Tyrosine-Tyrosine crosslinks within BSA-hydrogel samples. Triplicates of hydrogel samples with different AA concentrations (0%, 0.5%, 0.75%, 1%, and 1.5% (v/v)) were prepared as described previously. Each hydrogel sample was placed in a sealed Eppendorf tube of 1 mL HCl (6 N) and kept at 105 °C for 2 h, using a heat block to ensure full hydrolysis of amide bonds. Following treatment, 100 μL of acid hydrolysis product from each sample was transferred to a new 1.5 mL Eppendorf tube and neutralized by NaOH (5 M). After that, 0.1 M Na_2CO_3−$NaHCO_3$ buffer (pH ~ 9.9) was added to the neutralized solutions up to 1 mL final volume. The fluorescence emission intensity was recorded at 410 nm using the infinite M200 spectrophotometer (TECAN) plate-reader.

Mechanical Characterization. The mechanical characterization of the BSA and L8 - based hydrogel samples was performed by a force-clamp rheometer machine, as reported in our previous studies. Hydrogel samples were subjected to a force-ramp protocol with a controlled stress/relaxation rate of 0.01 mN/s (40 Pa/s) at room temperature while immersed in TRIS or 6 M GuHCl solution. Young's modulus was calculated from the slope over the elastic region of each stress-strain curve. The energy dissipation was calculated from the hysteresis area enclosed by the stress-strain curves.

Swelling ratio measurements. For water content experiments, triplicates of BSA-based hydrogels containing different concentrations of AA (0 – 1.5% (v/v)) were synthesized as described in the previous section and then soaked in TRIS buffer (20 mM and 150 mM NaCl, pH~7.4) at 4°C for 24 h. After that, the hydrogels were removed from the TRIS solution, blotted with filter paper to remove excess buffer, and then weighed to obtain each sample's wet weight (W_{wet}). Then, the same hydrogels were desiccated in a vacuum chamber for 24 h before weighing to obtain each sample's dry weight (W_{dry}). The swelling ratio was calculated using the following equation $\left(W_{wet} - W_{dry} \right) / W_{dry} \times 100$.

Cryo Scanning Electron Microscopy and Sample preparation. The hydrogel samples were placed and sandwiched between two aluminum discs (3 mm in diameter, 25μm in thick each) and cryo-immobilized in a high-pressure freezing device (EM ICE, Leica). The frozen samples were then mounted on a holder under liquid nitrogen in a specialized loading station (EM VCM, Leica) and transferred under cryogenic conditions (EM VCT500, Leica) to a sample preparation freeze fracture device (EM ACE900, Leica). In that device, the samples were fractured by a rapid stroke of a cryogenically cooled knife, exposing the inner part of the sandwiched discs. After fractured, the samples were etched at −100°C for 10 min to sublime ice from the sample surface and coated with 3 nm carbon. Samples were imaged in a Gemini SEM (Zeiss) by a secondary electron in-lens detector while maintaining an operating temperature of -120°C. The measurements were done at the Ilse Katz Institute for Nanoscale Science and Technology Ben-Gurion University of the Negev, Beer Sheva, Israel. Pore area analyses were characterized using ImageJ (NIH, USA).

Supplementary Figures

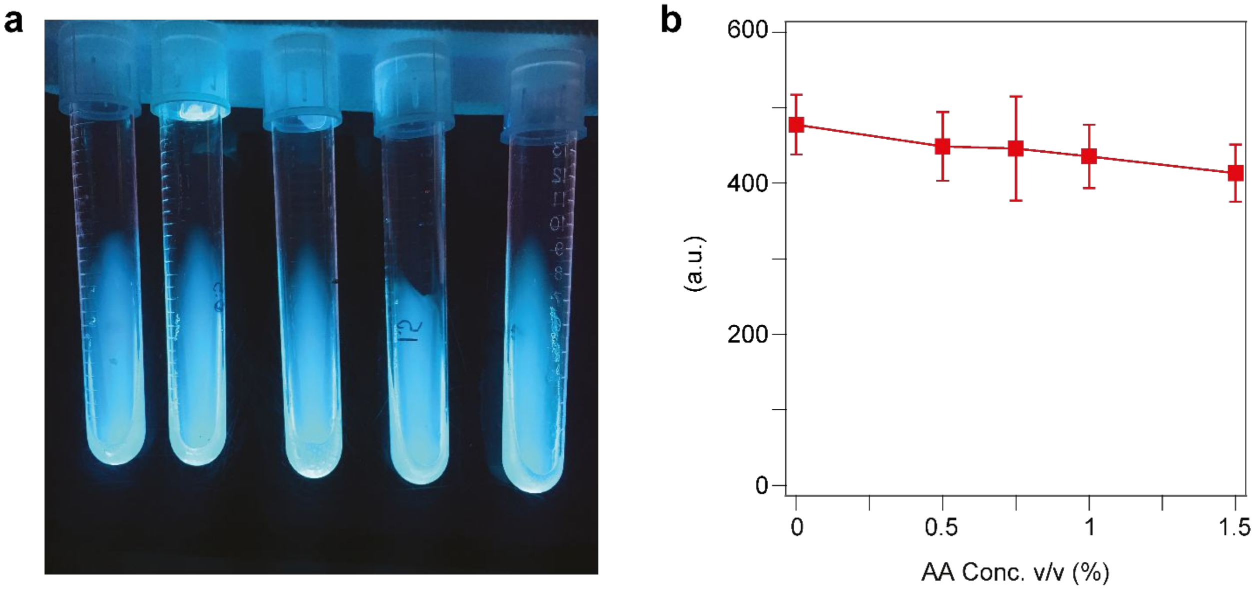

Supporting Figure 1. Examining the native structure of BSA in the presence of AA in the preparation buffer. (a) an image of BSA solutions with different AA concentrations incubated with ANS under UV light. (b) Fluorescence emission measurements of BSA/ ANS solutions as a function of AA concentration added to the solution. Measurements were collected after excitation at 470 nm. The constant fluorescence indicates that different concentrations of AA maintain the natural structure of BSA and allow ANS to bind.

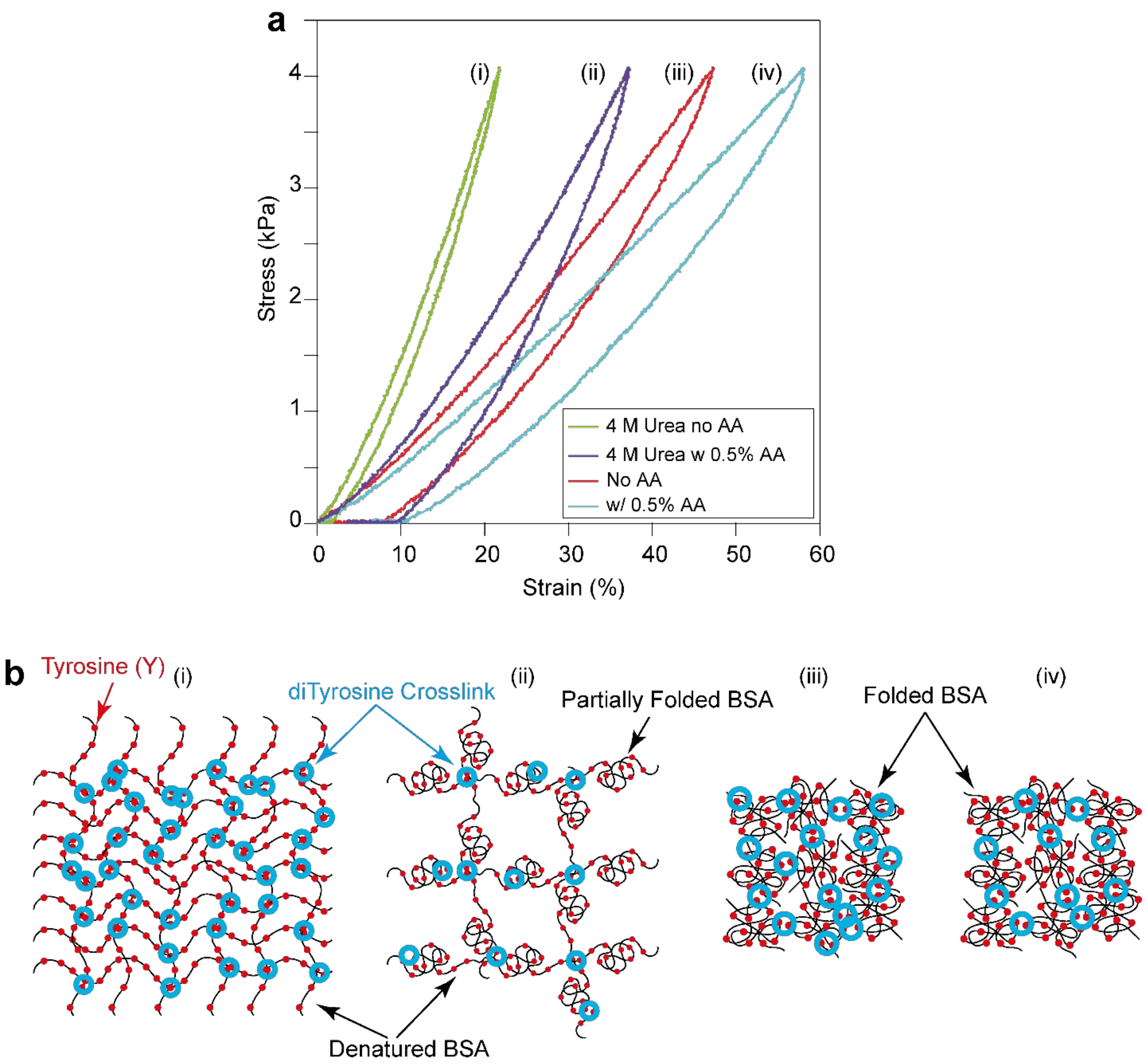

Supporting Figure 2. AA inhibits the formation of di-tyrosine crosslinks inside BSA-based hydrogels. (a) Stress-strain curves of (i) BSA-based hydrogel formed from BSA proteins that were denatured in 4 M UREA (ii) BSA-based hydrogel formed from BSA proteins that were denatured in 4 M UREA in the presence of 0.5% (v/v) AA in the preparation buffer. (iii) Native BSA-based hydrogel sample. (iv) Native BSA-based hydrogel sample with 0.5% AA in the preparation buffer. (b) A Scheme descri0bing the effect of AA on the hydrogel mechanical behavior. (i) Denatured BSA proteins crosslinked in the absence of AA. Due to BSA denaturing, more buried tyrosine

amino acids are exposed, causing an increase in crosslinking density, loss of protein folded structure, and loss of hysteresis, which translated into a stiffer hydrogel compared with native BSA-based hydrogel. (ii) Denatured BSA proteins crosslinked in the presence of 0.5% (v/v) AA. The AA decreases the crosslinking density during the photoactivated reaction, and the BSA domains fold partially after immersing the hydrogels in the TRIS buffer. This behavior is reflected by the decrease in Young's modulus and the slight increase in the hysteresis.

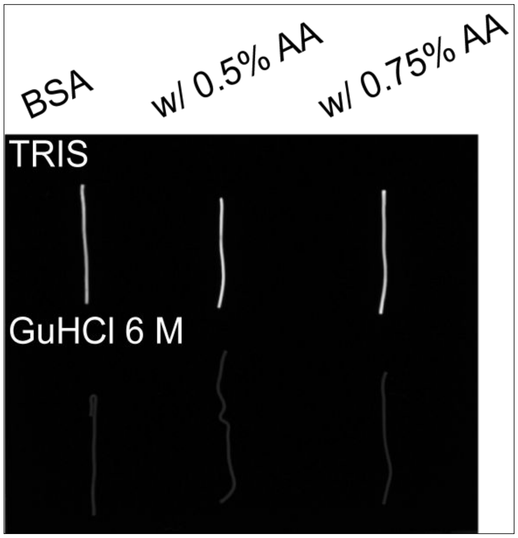

Supplementary Figure 3. Fluorescence image of BSA hydrogels prepared with different concentrations of AA immersed into TRIS and in GuHCl 6 M solution containing ANS. No significant change in the diameter of each sample was due to the addition of AA to the preparation buffer. Folded BSA hydrogel samples fluoresce under UV light (top line), while denatured BSA hydrogel samples lose their fluorescence.

To demonstrate that we can remove the crosslinking reagents from the hydrogel after the synthesis, we washed the hydrogel sample in TRIS solution three times. According to the FTIR spectra presented in Supplementary Figure 4, we succeeded in removing the APS and Ru (II) from the hydrogel sample as the characteristic bands of sulfate (~1049 cm^{-1}) in APS Supplementary Figure 4 (i) was suppressed in the spectra of the washed BSA-based hydrogel sample and remained only with the crosslinked BSA domains (Supplementary Figure 4 (iv) and (iii)).

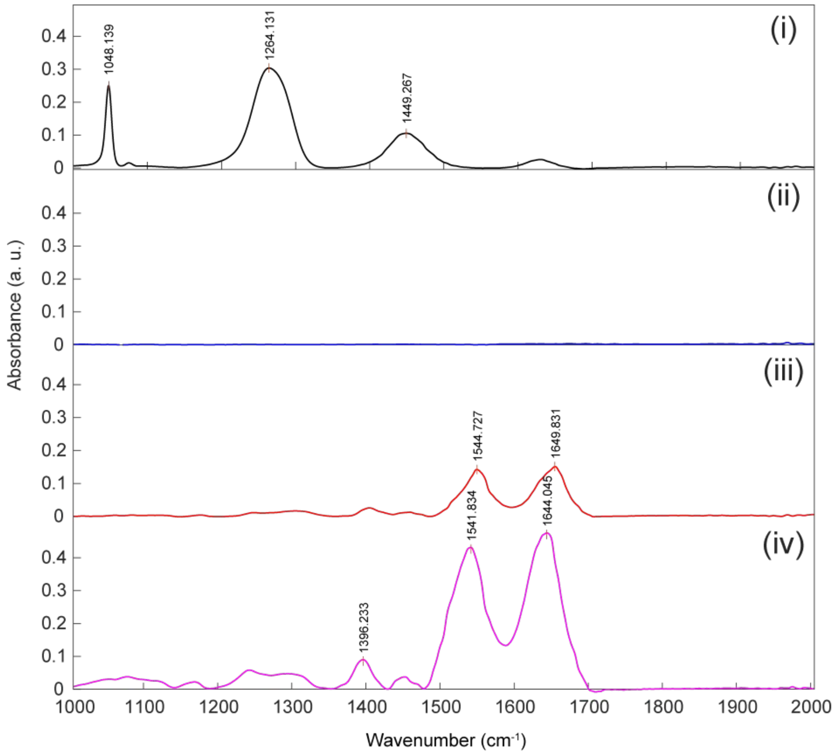

Supplementary Figure 4. FTIR spectra of (i) 1 M APS, (ii) 6.67 mM Ru (II), (iii) 2 mM BSA solution (iv) 2 mM BSA-based hydrogel after extensive wash with TRIS buffer.
